# Recommendations for improving the experimental protocol for the determination of photocatalytic activity by nitric oxide oxidation measurements

**DOI:** 10.55730/1300-0527.3612

**Published:** 2023-09-30

**Authors:** Selin ERNAM, Zeynep Ece AKGÜL, Deniz ÜNER

**Affiliations:** Department of Chemical Engineering, Faculty of Engineering, Middle East Technical University, Ankara, Turkiye

**Keywords:** Photocatalysis, nitric oxide oxidation, photocatalytic activity

## Abstract

The photocatalytic nitric oxide (NO) oxidation reaction is used as a standard diagnostic tool for photocatalytic activity according to the well-defined protocol described by ISO Standard 22197-1-2007. This protocol identifies the negative peak showing a NO_x_ concentration drop during a gas flow switch from the calibration bypass to the reactor as adsorption of NO_x_ on the surface. Evidence is provided for this first transient to be due to a dilution effect in the gas phase within the reactor. With proper models of residence time distribution analysis, this transient revealed the internal hydrodynamics and it can be used to determine the internal volumes of the system. The second transient occurs immediately after the light is switched on. The conversions strongly depend on the time constant of this transient. Controlled measurements of the effect of illumination intensity revealed that at higher light intensities the transient takes longer to reach steady state. The longer transient was attributed to the time needed to reach a thermal steady state of the hot spots generated by the recombination of excess charge carriers. When the catalyst amount was investigated as a parameter, a saturation effect was observed. This saturation effect was correlated with the gas phase concentrations of NO_x_ and moisture and their ratios to the available specific surface area. Hence, additional constraints with respect to the illumination intensity and catalyst amounts are recommended for accurate measurements of photocatalytic activity by NO oxidation.

## 1. Introduction

Photocatalytic nitric oxide (NO) oxidation is a standardized means of measuring the photocatalytic activity of a semiconductor photocatalyst according to the ISO-22197 standard [1]. This standard explains how the instruments should be set up and how to prepare a test piece, and it describes the experimental procedures. The measurement procedure is divided into three time-dependent key steps to ensure the accurate measurement of catalytic activity. The first of these time-dependent processes is for the preparation of the gases and calibration of the measurement device. The second is for establishing adsorption/desorption equilibrium with the gas phase and the test piece. The third is for the photocatalytic reaction. Thorough investigation of a semiconductor catalyst in this way can reveal much about photocatalytic reactions.

Many photocatalytic processes have bottlenecks in steps that are not specific to reactants but are rather common to photocatalytic processes such as charge carrier dynamics. Therefore, investigation of an arbitrary but easily measurable photocatalytic process such as NO oxidation can reveal much about the fundamental steps. This would lead to uncovering key unknowns about photocatalytic processes [2].

Impinging photons on semiconductors with a proper bandgap generates charge carriers. This is the first of the consecutive steps of photocatalytic reactions. Therefore, illumination intensity has substantial effects on photocatalytic reactions, as extensively investigated for both NO oxidation over TiO_2_ [3–6] and other photocatalytic reactions [7]. At low illumination intensities, the reaction rate linearly increases with increasing light intensity. As intensity increases, the relation between reaction rate and intensity becomes a square root relation, and further increase causes saturation and the reaction rate becomes independent of the illumination intensity [7]. However, to the best of our knowledge, there are no systematic studies analyzing the effects of catalyst amount.

TiO_2_ has been proven to have photoactivity towards the oxidation of NO. The transients in the process are also well known, but the reasons are not fully elucidated. This study reports the role of catalyst film thickness and illumination intensity in transient behavior during photocatalytic NO oxidation over TiO_2_ investigated in line with the ISO-22197-2007 standard protocol [1].

## 2. Materials and methods

### 2.1. Materials

Degussa P25 TiO_2_ from Evonik (Essen, Germany) was used as the catalyst. To coat TiO_2_ on a glass substrate, a slurry was prepared by mixing a known amount of powder with a known volume of distilled water. The aqueous mixture was uniformly coated on a glass substrate of 5 cm × 10 cm. The specimen prepared as such was dried under ambient conditions. The characterization data are reported in the supporting information as follows: Surface area measurements based on Brunauer–Emmett–Teller (BET) analysis are given in Table S1. The morphology of the material as determined by scanning electron microscopy is given in Figure S1. The crystallinity of the material is given in Figure S2. The defect concentrations measured by electron spin resonance (ESR) spectroscopy are given in Figure S3.

### 2.2. Catalyst characterization

The samples were analyzed by SEM (Vega 3 TESCAN, Brno, Czechia), X-ray diffraction (Rigaku Miniflex, 40 kV, CuK_α_, Rigaku, The Woodlands, TX, USA), and ESR spectroscopy (Bruker microESR, Bruker Corp., Billerica, MA, USA) before and after the measurements to ensure the morphological stability of the photocatalyst during reactions. The specific surface areas of these catalysts were characterized by BET method using nitrogen adsorption in the TriStar II system (Micromeritics, Norcross, GA, USA).

### 2.3. Photocatalytic NO oxidation measurement set-up

**Figure 1:**
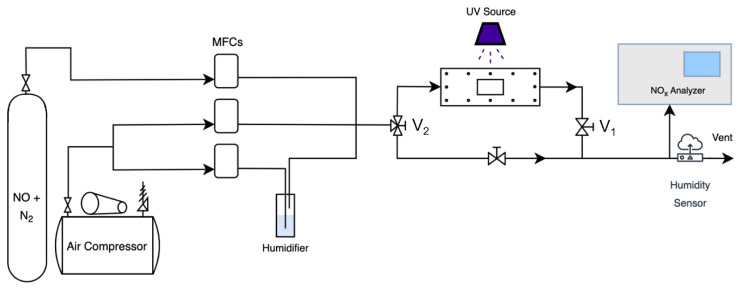
Schematic drawing of the NO oxidation set-up.

The NO oxidation measurement set-up consisted of an air compressor for a continuous supply of air, a gas cylinder containing a mixture of 100 ppm NO and nitrogen from Linde (Dublin, Ireland) for the test gas supply, three Teledyne (Thousand Oaks, CA, USA) HFC-202 mass flow controllers (MFCs), a humidifier for adjusting the humidity content of the test gas, a UV light transparent photoreactor, a UVP Blak-Ray B-100AP 250W 365 nm mercury UV lamp (Fisher Scientific, Waltham, MA, USA), and a model 42i NO_x_ analyzer (Thermo Scientific, Waltham, MA, USA). Figure 1 presents a schematic of the experimental set-up. Note that there are two lines for air, one of which goes through the humidifier. By adjusting the flow rates of gases flowing through the humidifier, the humidity content of the gas stream can be adjusted.

Photocatalytic NO oxidation experiments were conducted according to ISO Standard 221971-2007 [1]. With this protocol, a typical experiment is divided into three major steps. The procedure starts with the air + NO mixture flowing through the bypass line where the gas is sent directly to the NO_x_ analyzer. In this bypass stage, the NO concentration is adjusted from the MFC control station. When the desired inlet conditions are achieved, the flow is directed to the photoreactor by turning a three-way valve located before the reactor. When the flow is directed to the reactor, the gas is mixed with air in the extra volume of the reactor. This results in a dilution effect. The NO concentration drops; however, it recovers and reaches a steady state at its initially adjusted value quickly thereafter. This is observed as a negative peak in NO concentration versus time plots. The test gas is then allowed to flow through the reactor for 30 to 40 min without UV illumination, hence called the dark stage. The dark stage allows the gases to reach an adsorption/desorption equilibrium with the test piece. After 40 min, illumination is started and the reaction begins immediately. The initial conversion is high, but it gradually decreases as time progresses and eventually reaches a steady state. The experiment is stopped after steady state is achieved by switching off the UV illumination followed by NO flow shut-off and a 15-min degassing period is started. After the degassing period, the flow is directed back to the bypass stream from the reactor. Unless otherwise stated, the experiments of the present study were conducted at a flow rate of 1 L/min and 50% relative humidity under ambient conditions. The effects of illumination intensity, humidity, and catalyst amount on the time constant of the transient were investigated. The illumination intensity was varied by adjusting the distance between the UV source and the reactor. The UV light intensity was measured using a homemade UV light intensity measurement device that included a Grove UV light intensity sensor (Seeed Technology Co., Ltd., Shenzhen, China) and a data collection system specifically designed for this study.

### 2.4. Embedded systems and sensors

In this study, a commercial analog Grove UV light intensity sensor was used to measure UV light intensity and a digital DHT11 humidity and temperature sensor was used to measure the humidity of the gas stream. An mbed-enabled Freedom K64F microcontroller board was used to acquire serial data from the sensors. The K64F pinout is similar to that of the Arduino R3, a commonly available microcontroller board, making the K64F compatible with many shields and other prototyping components designed for the Arduino R3. A Grove base shield was used for easy prototyping. Sensors and other components can be connected to the microcontroller board via the shield with universal four-pin connector cables. In this study the analog, digital, and I2C pins were used for the analog and digital sensors and an I2C-interfaced LCD, respectively.

The microcontroller board can communicate with a computer serially via a cable and information such as sensor readings and time can be printed on a screen via a terminal emulator. However, a computer may not always be available near the measurement site, which introduces difficulty in taking measurements. A portable device on which data can also be displayed is convenient for these situations. A small liquid crystal display was used for this purpose. A Grove RGB LCD with I2C interfacing was used to display sensor readings and a clock when necessary. I2C is a serial communication protocol where only two data wires are necessary, making it easy to use for such applications [8]. The LCD module is therefore easily attached with a four-pin connector to the I2C port on the base shield. With this setup, only a 5-V DC power supply is necessary for measurements to take place [8].

### 2.5. Light intensity adjustment and measurement

**Figure 2:**
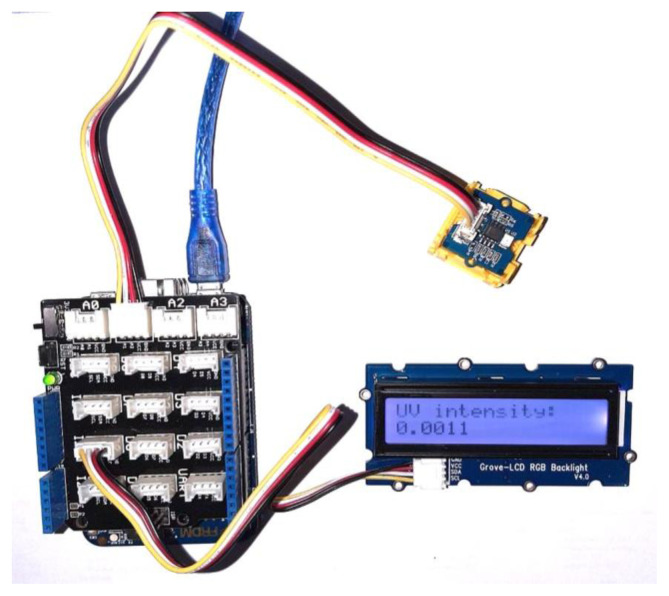
UV light intensity sensor with K64F microcontroller board with a base shield.

The UV light intensity sensor and the microcontroller board can be seen in Figure 2.

The main parameter of this study is the effect of light intensity on photocatalysis. The light intensity during experiments was increased or decreased by adjusting the distance between the source and the reactor using a height-adjustable stand. To achieve lower illumination intensities, a black carton shade with slits was used. The UV sensor was placed on the photocatalytic reactor to measure the incident light intensity. Details of the verification and calibration can be found in the supplementary information (Figures S4 and Figure S5).

## 3. Results and discussion

**Figure 3:**
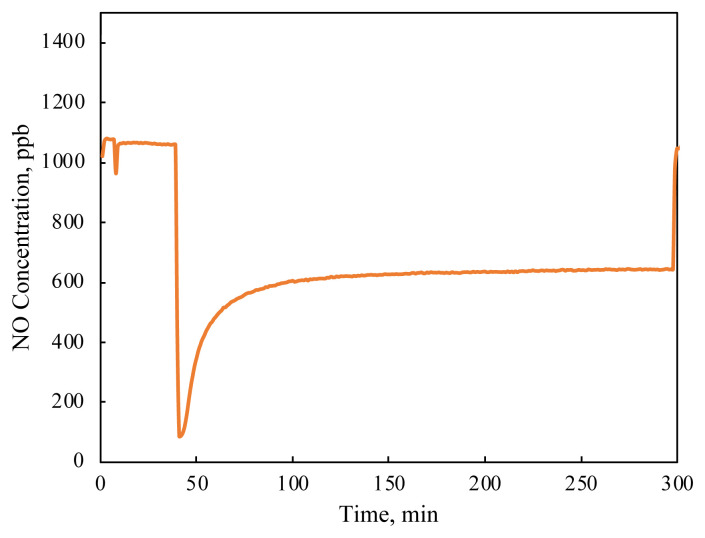
A typical NO oxidation measurement of the TiO_2_ specimen. Measurement conditions are 35 mW/cm^2^ illumination intensity, 30% RH.

The test protocol requires activation and surface cleaning by a flow of humid air, preferably in the presence of photon flux. Then, before introducing the reactive gases, the light is turned off and the gas flow is diverted to a bypass for the calibration of the NO_x_ analyzer with respect to the concentration of the incoming stream. Under the gas calibration sequence, the reactor is filled with humid air without any NO_x_, while the stream with NO_x_ is flowing through a bypass line to the analyzer. Once the calibration is completed, the flow is redirected to the reactor. This is the second period in the protocol, ensuring that the catalyst surface is saturated with the moisture, oxygen, and NO_x_ gases. Immediately upon the gases being redirected to the reactor, a negative peak appeared in the analysis, which quickly recovered back to the feed concentrations as can be seen in Figure 3. This feature is attributed to adsorption in the ISO standard. However, the decrease in signal intensity may be due to factors other than adsorption for two reasons. First of all, it is widely known that NO gas does not adhere to the surface well [9–13]. Second, and more importantly, the gas stream containing NO_x_ is introduced to a reactor which is filled with humid air with no NO_x_ gases, and so a dilution followed by a breakthrough process is more likely to be the reason for this negative peak.

### 3.1. Transient material balance around the reactor during the dark period

The residence time distributions were measured in an empty reactor as a function of the inlet tracer concentration and the flow rate and are given in Figure S6 in the supplementary information. The appropriate conditions to provide the needed mixing conditions were selected based on guidance obtained from the data presented in Figure S6. The residence times of the gas in the reactor, the volume of the reactor space, and the concentration of the feed gas can all be inferred from the negative peak. A transient mass balance for an inert tracer given to a flow reactor can be written as follows:


(1) $$\frac{dN}{dt}=F_{in}-F_{out},\;\;\;\text{subject to the following initial condition: at t}=0,\text{N}=0$$

In this mole balance equation, N represents the tracer amount within the system boundaries, while F represents the molar flow rates in and out of the reactor. The initial condition indicates that initially the reactor has no tracer molecules. Once the gases are diverted to the reactor, the inlet concentration will be known and constant, while the outlet concentration will be a function of time until the reactor is completely filled with the incoming gas.

The molar flow rates can be written in terms of the volumetric flow rates and the concentrations of the incoming streams as follows:


(2) $$F_{in}=v_{0}\;C_{in},F_{out}=v_{0}\;C_{out},$$

Similarly, it is possible to redefine N in Eq. (1) as N = VC_out_, where V is the gas holdup volume of the reactor and C_out_ is the time-dependent concentration. With these substitutions, Eq. (1) can be reorganized as follows:


(3) $$\tau \frac{dC_{out}}{dt}=C_{in}-C_{out}\;\;\;\text{at t}=0,{\text{C}}_{\text{out}}=0$$

The balance is written by assuming a certain degree of mixing in the reactor. In fact, the whole purpose of this analysis is to determine the degree of mixing by time-dependent tracer concentration. In Eq. (3), the new parameter τ is introduced, defined as as the space time or residence time of the fluid in the reactor.

A tank in the series model is used to write the transient mass balance. The regions from valve V2 to V1, including the reactor volume (tank 1) and valve V1 to the NO_x_ analyzer (tank 2), are assumed to be two well-mixed tanks connected in series. The modeling starts at the instant of the redirection of the flow from the bypass to the reactor. Immediately after the flow is redirected to the reactor, the incoming gases are diluted with the tracer-free gas filling the reactor. Noting that the pipeline after valve V1 is filled with NO_x_-containing gas due to calibration, one would expect a dilution only due to the reactor volume. As a result, the time dependency of the NO_x_ concentration will reveal both information about the effective reactor volume and also how effective the gas-phase mixing characteristics are. The model requires different initial conditions for each tank. Since the reactor is initially filled with air only, the initial concentration of tank 1 is zero. The pipeline after V1 being filled with calibration gas causes the initial concentration of tank 2 to be equal to the given tracer concentration, C_0_.

The exit concentration of tank 1 is time-dependent and expressed as C_1_. The transient mass balance for tank 1 is as follows:


(4) $$\tau_{1}\frac{dC_{1}}{dt}=C_{0}-C_{1}$$

The solution of the ordinary differential equation reveals the following concentration profile:


(5) $$C_{1}=C_{0}(1-e^{\frac{t}{\tau_{1}}})$$

Using C_1_ as the inlet concentration of tank 2, a new transient mass balance is written for the second tank. C_2_ represents the exit concentration of the second tank, which is directly sent to the NO_x_ analyzer.


(6) $$\tau_{2}\frac{dC_{2}}{dt}=C_{0}-C_{1}$$

The concentration profile of the exit of tank 2 was modeled as follows:


(7) $$C_{2}=C_{0}-\frac{C_{0}\tau_{1}}{\tau_{1}-\tau_{2}}\;(e^{-\frac{t}{\tau_{1}}}-e^{-\frac{t}{\tau_{2}}})$$

**Figure 4:**
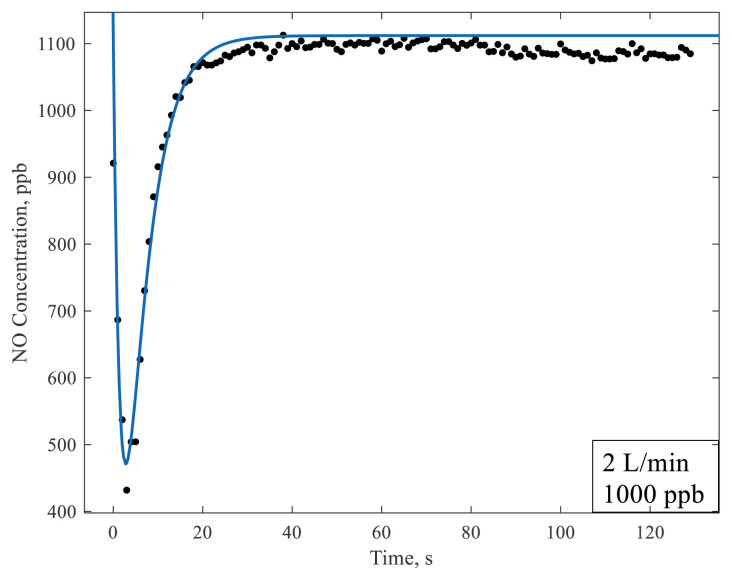
The data with a comparison to the model prediction to estimate the residence times of the gas through the reactor. The gas flow rate was 2 L/min, while NO_x_ concentration of 1000 ppb was used for this experiment.

To analyze the negative peak mentioned in the previous section in detail, the sampling frequency was increased from per minute to per second. The experimental data were fitted to the concentration profile, C_2_, to obtain τ_1_ and τ_2_ using the MATLAB Curve Fitter (MathWorks, Natick, MA, USA). The concentration profiles obtained from the mass balances are plotted together with the experiment in Figure 4.

The space time estimated for the hypothetical tank 1 is 5.1 s, while that of hypothetical tank 2 is estimated as 1.7 s for the total NO volumetric flow rate of 2 × 10^−3^ mL/min. These hypothetical tanks represent the reaction chamber and the transfer line between the reactor exit and the sensor, respectively.

With the analysis provided above, it was demonstrated that the decrease in concentration was due to a dilution and recovery effect after the gas flow was diverted from the bypass to the reactor [2,14]. This analysis was based on the fact that the effect of NO adsorption is weak on the transient. This is only possible if the NO adsorption is not the prevailing process under the experimental conditions. Among many supporting arguments that NO adsorption on TiO_2_ is weak, here the data reported by Yates et al. in 2000 were used [13]. Yates et al. [13] showed with temperature-programmed desorption that NO adsorbs weakly on oxidized TiO_2_ and desorbs at 127 K. Their data for different species were estimated as a result of both theory and experiment and are given in the Table [13].

**Figure 5:**
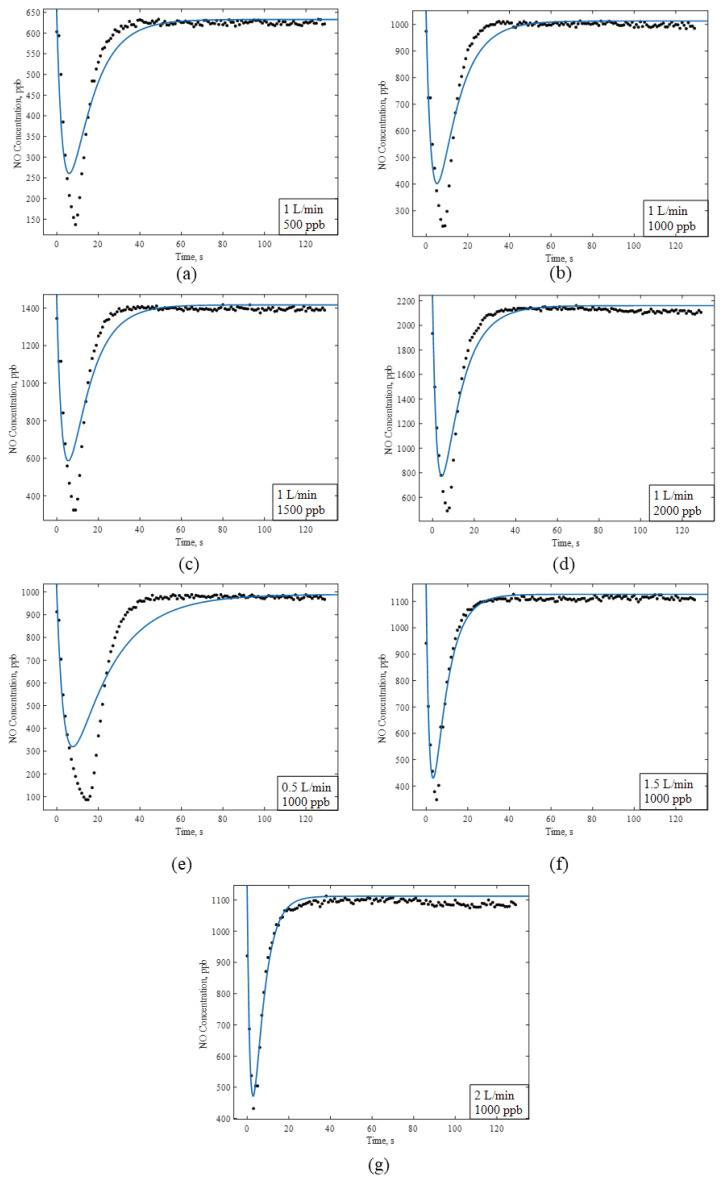
Data and model predictions for 1 L/min flow rate and NO concentrations of (a) 500 ppb, (b) 1000 ppb, (c) 1500 ppb, and (d) 2000 ppb, as well as for 1000 ppb inlet concentration and (e) 0.5 L/min, (f) 1.5 L/min, and (g) 2 L/min flow rates.

In order to demonstrate the effects of concentration and flow rate on the behavior of the reactor, data collected by varying the inlet NO concentrations and flow rates were compared against the estimations of the tanks in the series model. The results of the data and the model predictions are shown in Figure 5. The Curve Fit feature of MATLAB was used to estimate the space times, which are the adjustable parameters of the model given in Eq. (7). The estimated R-square values varied between 0.85 and 0.96, decreasing with increasing flow rate. In other words, as the flow rate increased, the reactor exhibited better mixing performance.

**Figure 6:**
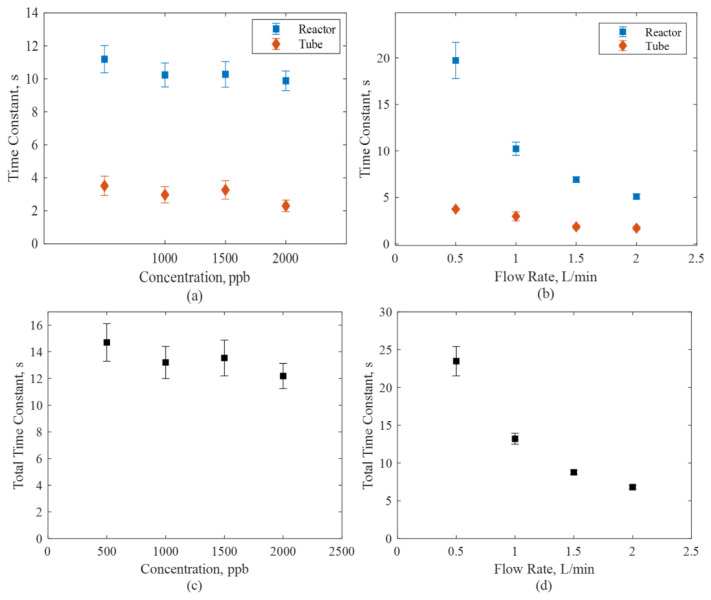
Time constants of various initial concentrations (a) individually and (c) in total, and for various flow rates (b) individually and (d) in total.

Figure 6 shows the estimated space times of tank 1 and tank 2 for the model predictions in Figure 5. Tank 1 and tank 2 are represented as a reactor and a tube, respectively. Since tank 1 involves the reactor volume, the time constants of tank 1 are higher than those of tank 2, as expected.

The time constant analysis shown in Figure 6 consistently reveals that the effect of concentration on the time constant is minimum, as expected. On the other hand, the time constant decreases linearly with increasing flow rate, also indicating that the mixing characteristics in the reactor are adequate. It should be noted that all the flow regimes were laminar with Reynolds numbers of 19.3, 38.6, 57.9, and 77.2 for flow rates of 0.5, 1, 1.5, and 2 L/min, respectively.

### 3.2. Effect of illumination intensity

**Figure 7:**
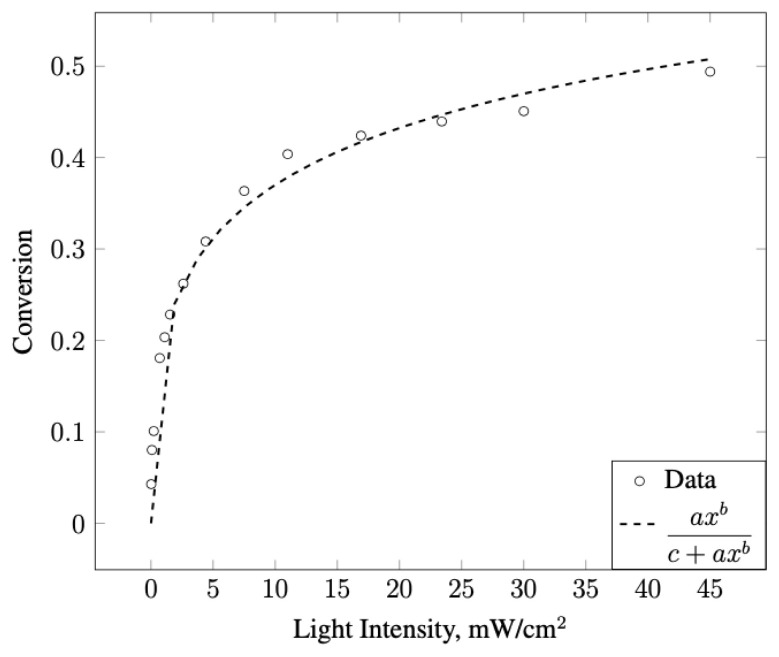
NO conversion at steady state with respect to illumination intensity and curve fit, where a = 0.1809, b = 0.3733, c = 0.7265, and R^2^ = 0.992.

The effect of illumination intensity on the characteristic transient and steady-state conversion was investigated. Upon illumination, a high initial conversion was achieved, which gradually evolved to a lower steady-state conversion value. This transient is not observed at low illumination intensities. There is a direct correlation between the illumination intensity and the steady-state conversion, consistent with other studies [7]. Steady-state conversions under different illumination intensities were measured (see Figure S7 in the supplementary information) and the relationship between the illumination intensity and NO conversion is shown in Figure 7. The nonlinear behavior of the data shown in Figure 7 can be attributed to a saturation effect at higher illumination intensities. The curve fit revealed the relation between conversion and light intensity for our experiments, as given in Figure 7.

### 3.3. Effect of catalyst amount

**Figure 8:**
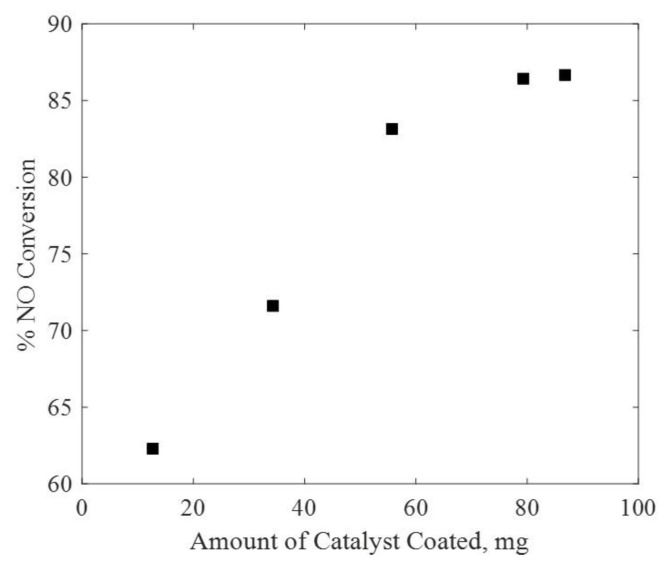
Steady-state NO conversion with respect to amount of catalyst coated at 45 mW/cm^2^.

**Figure 9:**
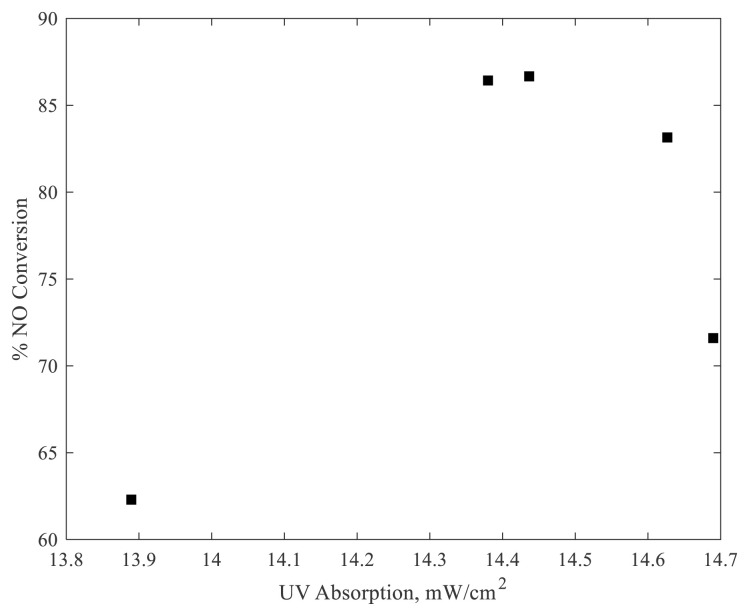
Steady-state NO conversion with respect to absorbed light.

To investigate the effect of catalyst coating, five different test pieces were coated with varying amounts of TiO_2_ (12.7 mg, 34.3 mg, 55.7 mg, 79.3 mg, and 86.8 mg). The experiments were conducted at light intensity of 45 mW/cm^2^. As shown in Figure 8, with increasing catalyst amounts, the steady-state NO conversion increases. However, the deactivation behavior of the system is less pronounced at higher catalyst loadings (see Figures S8 and Figure S9 in the supplementary information. For 34.3 mg of titania, the initial NO concentration increased unexpectedly to levels of about 1200 ppb. Even though the reason for this was unclear, it is not expected to have played a role in the results. From Figure 8, it can be observed that the steady-state conversion values are closer to each other at higher catalyst loadings, suggesting a saturation effect. For each test piece, the transmitted light intensity was therefore measured and the absorbed light intensity was calculated. The steady-state NO conversion as a function of absorbed light intensity can be seen in Figure 9.

The data shown in Figure 9 clearly reveal a maximum in NO conversion as a function of the absorbed light intensity. Further increase in the light intensity decreased the NO conversion, most probably due to the thermal effects arising from the exothermic recombination of the excess charge carriers [15,16]. At higher local temperatures, the adsorbed amounts of water, oxygen, and NO_x_ will decrease, leading to decreased NO_x_ conversions.

As a final note, Figure S10 provides direct evidence that the initial negative peak observed in NO concentration under dark conditions is not due to adsorption. It is due to a dilution effect caused by a bypass process. This is a natural consequence of the transfer of the NO-laden gas from the bypass stream to the reactor. The peak does not show any dependence on the catalyst amount; it is only a function of the flow rate, as can be seen in Figure S10.

## 4. Conclusions

Photocatalytic NO oxidation experiments are used to determine the photocatalytic activity of catalysts based on standard protocols such as ISO Standard 22197-1-2007. As a result of this study, several recommendations can be made to improve this standard protocol as follows. First of all, the initial negative peak observed in NO concentration during these measurements is not due to adsorption; rather, it is due to a dilution and refill process that is a natural consequence of the transfer of the NO-laden gas from the bypass stream to the reactor. The second recommendation is to measure the light intensity that is absorbed (or transmitted) by the catalyst in order to avoid oversaturation of the surface with photons. Oversaturation has a potential to create excess charge carriers that will undergo exothermic recombination, creating local hot spots and causing premature desorption of adsorbed molecules, which in turn will lead to a decrease in NO conversion. The other parameter that needs to be optimized is the catalyst amount. A rigorous analysis of the activity should be reported per gram of catalyst. The catalyst amount and the incoming NO_x_ concentration must be commensurate for a correct assessment. Low and insufficient catalyst amounts may lead to underestimation of the conversion, and a correct comparison requires finding the amount of catalyst for which, upon further addition, NO_x_ conversion does not improve. Furthermore, the initial transient upon illumination has a number of dynamic processes taking place simultaneously. Sufficient time must be allotted for the system to evolve to a true steady state.

## Supplementary information

The photocatalytic material used in this study was thoroughly characterized by SEM, XRD, and EPR spectroscopies before and after use. In this section, these results are presented.

### SEM images

The SEM images of three different TiO_2_ coated samples are shown in Figure S1. Figure S1a is fresh TiO_2_ P25 powder, Figure S1b is from the catalyst test piece used for NO oxidation measurements, and Figure S1c is an image of TiO_2_ P25 powder mixed with water and then dried to simulate the coating process. When fresh TiO_2_ P25 powder is mixed with water and later dried, the porous structure changes. Particles are clustered and chunkier in Figures S1b and S1c.

### BET analysis

BET analysis results are presented in Table S1. As can be seen, the specific surface area of the sample did not change appreciably due to the process requiring a slurry preparation with subsequent drying.

**Table S1:** BET surface areas of TiO_2_ used in this study.

Sample	BET surface area (m ^2^/g)
As-received TiO _2_	49.4
TiO _2_ dried from a slurry	51.8

Figure S1 SEM images. [a] is fresh TiO_2_. [b] is used catalyst obtained from the test piece. [c] is dried TiO_2_ from a slurry.

### XRD analysis

The XRD analysis results of fresh TiO_2_ P25 and dried TiO_2_ slurry are shown in Figure S2. The anatase/rutile ratio was found to be 81% anatase phase from peak analysis as described by Myers et al. [1]. The anatase/rutile ratio was not influenced appreciably due to drying from a slurry, confirming that the properties of the coated catalyst are similar to those of the as-received material.

Figure S2 XRD patterns of fresh and dried slurry TiO_2_ P25 powder.

### ESR spectroscopy

The ESR spectra of two samples are shown in Figure S3. The samples were respectively fresh and used TiO_2_. The samples were measured both under ambient conditions and under vacuum. The details of the vacuum manifold can be found in [2]. The EPR signal obtained under vacuum had features suggesting the presence of oxygen vacancies [3]. Due to the low signal-to-noise ratio of the data that could be obtained from a benchtop unit, further elaboration was not possible. However, distinct differences were observed between the as-received and used materials, suggesting the direct participation of TiO_2_ in the NO oxidation process, most probably by oxygen vacancy formation.

Figure S3 ESR spectra obtained from fresh and used catalyst. Measurements under vacuum are also shown.

### Calibration of the light intensity sensor

The details of the calibration of the light intensity sensor are explained in the MSc thesis of Ernam [4] and are briefly discussed in this section. The illumination intensity was adjusted by varying the height difference between the reactor and the light source. Light intensities at various positionings of the light source were measured using a commercial UV intensity sensor. Two measurement coordinates, height and base distance, were varied. Height corresponds to the vertical distance between the light source and the sensor. Base distance corresponds to the horizontal distance from the base of the light source and the sensor. The two measurement coordinates are shown in Figure S4. Light intensities measured with respect to height and base position are reported in Table S2. The illumination intensity at a base distance of 10 cm with respect to height is plotted in Figure S5.

Figure S4 UV light intensity measurement set-up schematic [4].

Figure S5 UV light intensity at base distance of 10 cm [4].

**Table S2:** Light intensities with respect to height and base distance.

Height (cm)	Base (cm)	Intensity (mW/cm ^2^)
0	0	0.18
0	5	2.95
0	10	23.9
0	15	35.4
10	0	0.84
10	5	2.63
10	10	9.8
10	15	33.5
15	0	0.76
15	5	1.47
15	10	5.37
15	15	14.23
20	0	0.4
20	5	1.46
20	10	4.64
20	15	12.16
30	0	0.63
30	5	0.8
30	10	2.19
30	15	4.31
40	0	0.57
40	5	0.87
40	10	1.73
40	15	3.478
50	0	0.51
50	5	0.77
50	10	1.32
50	15	2.56
50 cm + filter	0	0
50 cm + filter	5	0
50 cm + filter	10	0
50 cm + filter	15	0.27

### Time constant calculations

Time constant calculations are given below.

Mass balance around tank 1:


$$\begin{array}{l} \frac{{\text{dN}}_{1}}{\text{dt}}=\dot{\text{V}}({\text{C}}_{0}-{\text{C}}_{1})\\ {\text{V}}_{1}\frac{{\text{dC}}_{1}}{\text{dt}}=\dot{\text{V}}({\text{C}}_{0}-{\text{C}}_{1})\\ \tau_{1}\frac{{\text{dC}}_{1}}{\text{dt}}=({\text{C}}_{0}-{\text{C}}_{1})\\ \frac{{\text{dC}}_{1}}{\left({\text{C}}_{0}-{\text{C}}_{1}\right)}=\frac{1}{\tau_{1}}\text{dt}\\ \int_{0}^{{\text{C}}_{1}}\frac{{\text{dC}}_{1}}{\left({\text{C}}_{0}-{\text{C}}_{1}\right)}=\int_{0}^{\text{t}}\frac{1}{\tau_{1}}\text{dt}\\ -\text{ln}\frac{{\text{C}}_{0}-{\text{C}}_{1}}{{\text{C}}_{0}}=\frac{\text{t}}{\tau_{1}}\\ {\text{C}}_{1}={\text{C}}_{0}(1-{\text{e}}^{-\frac{\text{t}}{\tau_{1}}}) \end{array}$$

Mass balance around tank 2:


$$\begin{array}{l} \frac{{\text{dN}}_{2}}{\text{dt}}=\dot{\text{V}}({\text{C}}_{1}-{\text{C}}_{2})\\ \tau_{2}\frac{{\text{dC}}_{2}}{\text{dt}}=({\text{C}}_{1}-{\text{C}}_{2}) \end{array}$$

C_1_ is replaced with the result of the mass balance around tank 1:


$$\begin{array}{l} \tau_{2}\frac{{\text{dC}}_{2}}{\text{dt}}=({\text{C}}_{0}(1-{\text{e}}^{-\frac{\text{t}}{\tau_{1}}})-{\text{C}}_{2})\\ \frac{{\text{dC}}_{2}}{\text{dt}}+\frac{{\text{C}}_{2}}{\tau_{2}}=\frac{{\text{C}}_{0}}{\tau_{2}}(1-{\text{e}}^{-\frac{\text{t}}{\tau_{1}}}) \end{array}$$

Multiplying each side by :


$$\begin{array}{l} \frac{\text{d}}{\text{dt}}\left({\text{C}}_{2}*{\text{e}}^{\frac{\text{t}}{\tau_{2}}}\right)=\frac{C_{0}}{\tau_{2}}\left(1-{\text{e}}^{-\frac{\text{t}}{\tau_{1}}}\right)*{\text{e}}^{\frac{\text{t}}{\tau_{2}}}\\ {\text{C}}_{2}*{\text{e}}^{\frac{\text{t}}{\tau_{2}}}=\frac{{\text{C}}_{0}}{\tau_{2}}\left(\tau_{2}*{\text{e}}^{-\frac{\text{t}}{\tau_{2}}}-\frac{\tau_{1}\tau_{2}}{\tau_{1}-\tau_{2}}*{\text{e}}^{\frac{\tau_{1}\tau_{2}}{\tau_{1}-\tau_{2}}}\right)+\text{in}\\ {\text{C}}_{\text{2}}={\text{C}}_{0}-\frac{{\text{C}}_{0}\tau_{1}}{\tau_{1}-\tau_{2}}*{\text{e}}^{-\frac{\text{t}}{\tau_{1}}}+\lambda *{\text{e}}^{-\frac{\text{t}}{\tau_{2}}} \end{array}$$

At t = 0 and as t goes to infinity, C = C_0_:


$$\begin{array}{l} \lambda =\frac{{\text{C}}_{0}\tau_{1}}{\tau_{1}-\tau_{2}}\\ {\text{C}}_{\text{2}}={\text{C}}_{0}-\frac{{\text{C}}_{0}\tau_{1}}{\tau_{1}-\tau_{2}}*{\text{e}}^{-\frac{\text{t}}{\tau_{1}}}+\frac{{\text{C}}_{0}\tau_{1}}{\tau_{1}-\tau_{2}}*{\text{e}}^{-\frac{\text{t}}{\tau_{2}}}\\ {\text{C}}_{\text{2}}={\text{C}}_{0}-\frac{{\text{C}}_{0}\tau_{1}}{\tau_{1}-\tau_{2}}({\text{e}}^{-\frac{\text{t}}{\tau_{1}}}-{\text{e}}^{-\frac{\text{t}}{\tau_{2}}}) \end{array}$$

MATLAB curve-fitting results

**Table 4:** Goodness-of-fit for different concentrations

	0.5 L/min	1 L/min	1.5 L/min	2 L/min
SSE	1.4062e+06	3.8323e+05	1.1722e+05	1.0258e+05
R-square	0.8473	0.9089	0.9625	0.9487
DFE	128	128	128	128
Adj R-sq	0.8461	0.9082	0.9622	0.9483
RMSE	104.8145	54.7175	30.2621	28.3092

**Table 5:** Goodness-of-fit for different flow rates

	500 ppb	1000 ppb	1500 ppb	2000 ppb
SSE	1.7640e+05	3.8323e+05	8.6716e+05	1.2302e+06
R-square	0.8988	0.9089	0.8927	0.9334
DFE	128	128	128	128
Adj R-sq	0.8980	0.9082	0.8919	0.9329
RMSE	37.1226	54.7175	82.3085	98.0371

### RTD analysis

To describe the behavior of the reactor under different conditions, RTD analysis was also performed. NO was used as a tracer and was supplied to the system as a step input. NO concentrations and inlet flow rates of the gas mixture were varied to observe the changes in system dynamics. Figure S6 shows the system responses for different flow rates and concentrations. As the flow rate increased, the behavior of the system approached that of a continuous stirred tank reactor. In other words, the mixing performance of the system increased.

As the catalyst weight increases, the required time for the NO_2_ concentration to reach a steady state also increases. This behavior may be explained with the adsorption of NO_2_ and HNO_3_ to the surface as nitric acid is formed according to the reactions given below:


$$\begin{array}{l} 4\;{HNO}_{3}↔4{NO}_{2}+O_{2}+2H_{2}O\\ 3{NO}_{2}+H_{2}O↔2{HNO}_{3}+NO \end{array}$$

Figure S6 RTD analysis for various (a) tracer concentrations and (b) flow rates.

Figure S7 Effect of UV light intensity on NO oxidation raw data for the correlation shown in Figure 7.

Figure S8 Effect of catalyst coating on NO oxidation at 45 mW/cm^2^. Raw data for the correlation shown in Figure 8.

Figure S9 Effect of catalyst coating on NO_2_ formation at 45 mW/cm^2^.

Figure S10 Effect of catalyst coating on first transient at 45 mW/cm^2^. Data were averaged every minute. It can be seen that peak intensities do not vary with catalyst amount.

References for supplementary information1
SpurrRA
MyersH
Quantitative analysis of anatase-rutile mixtures with an X-ray diffractometerAnalytical Chemistry1957297607622
UnerD
TapanNA
OzenI
UnerM
Oxygen adsorption and spillover over Pt/TiO_2_ catalystsApplied Catalysis A: General20032512252343
ÜnerD
YararM
Oxygen vacancies on Pd/TiO_2_ are detected at low pressures by ESR spectroscopy at ambient temperaturesTurkish Journal of Chemistry2022461081108810.55730/1300-0527.341637538751PMC103956774
ErnamS
Investigation of the transient behaviors of photocatalytic NO oxidation over TiO2MScMiddle East Technical UniversityAnkara, Türkiye2022

## Figures and Tables

**Table:** Adsorption energies of NO_x_ species [13].

Species	Adsorption energy (kJ/mol)
NO	35.1
N_2_O	32.3
NO_2_	8.8
