# Supplementary figures and images for: Recommendations for improving the experimental protocol for the determination of photocatalytic activity by nitric oxide oxidation measurements
Source: Turk J Chem. 2023 Sep 30;47(5):1285–95. doi: 10.55730/1300-0527.3612 (PMC10760864; doi:10.55730/1300-0527.3612)

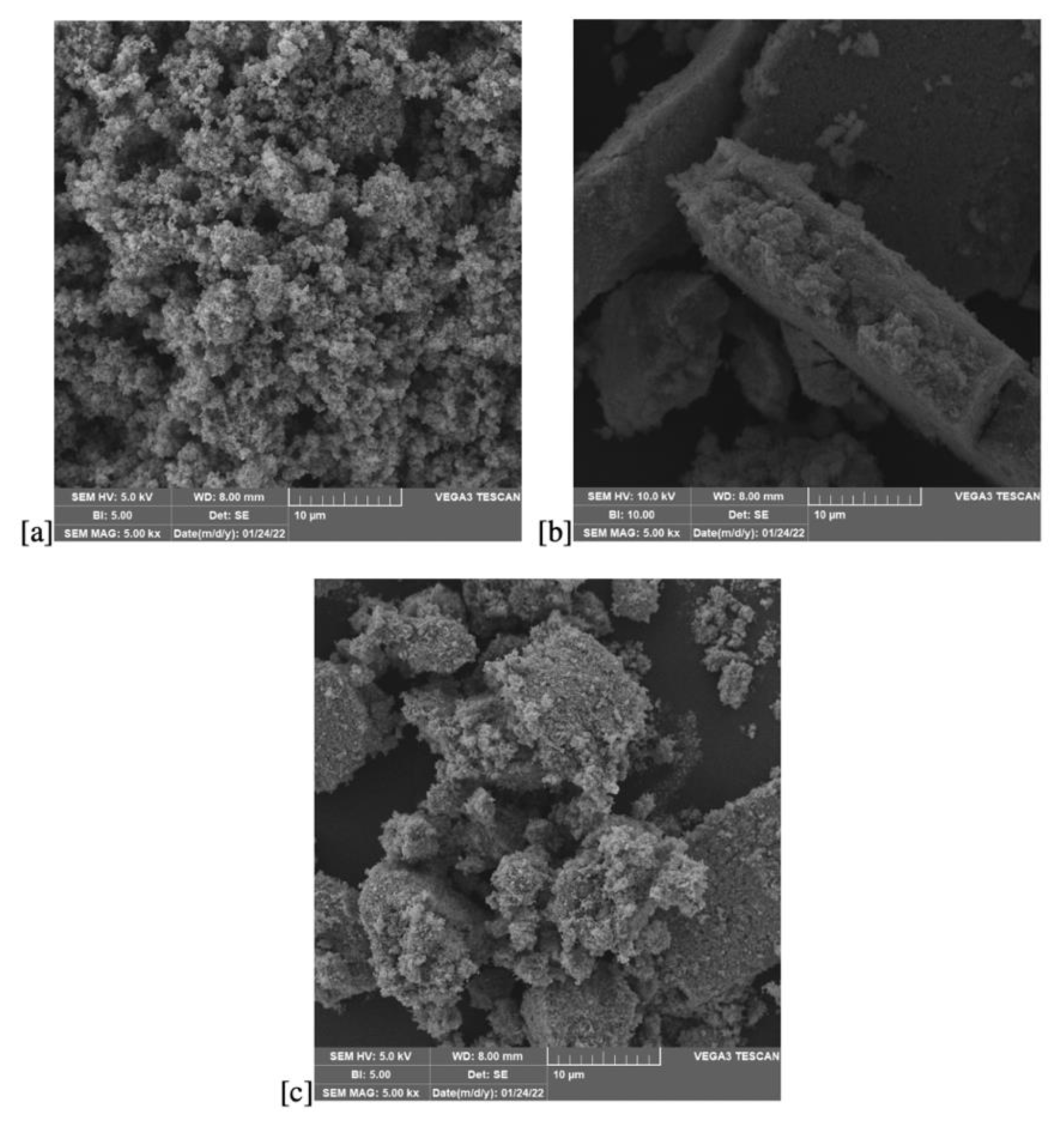

Supplement: Figure S1 — SEM images. [a] is fresh TiO2. [b] is used catalyst obtained from the test piece. [c] is dried TiO2 from a slurry. [file turkjchem-47-5-1285s1.tif]

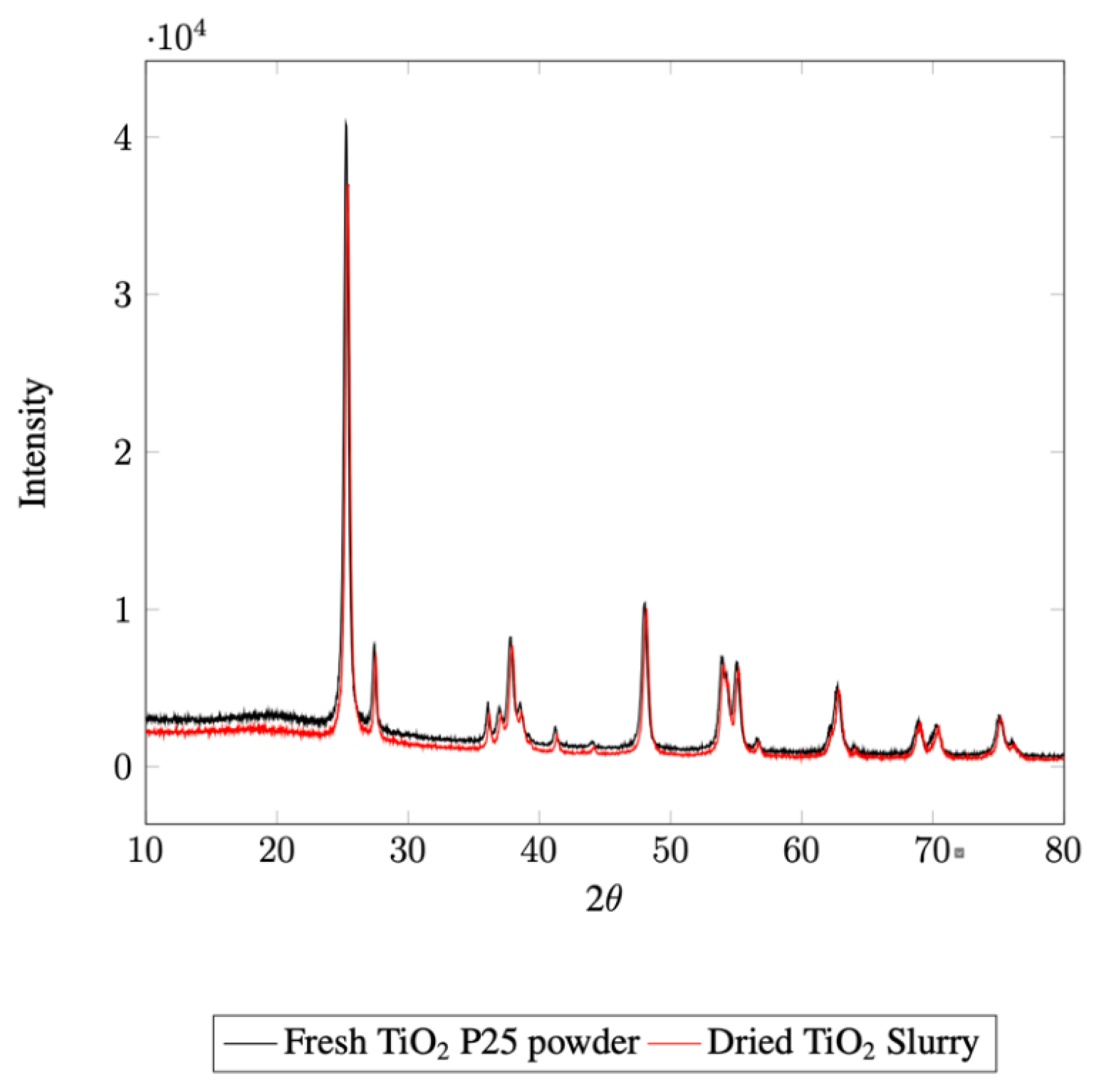

Supplement: Figure S2 — XRD patterns of fresh and dried slurry TiO2 P25 powder. [file turkjchem-47-5-1285s2.tif]

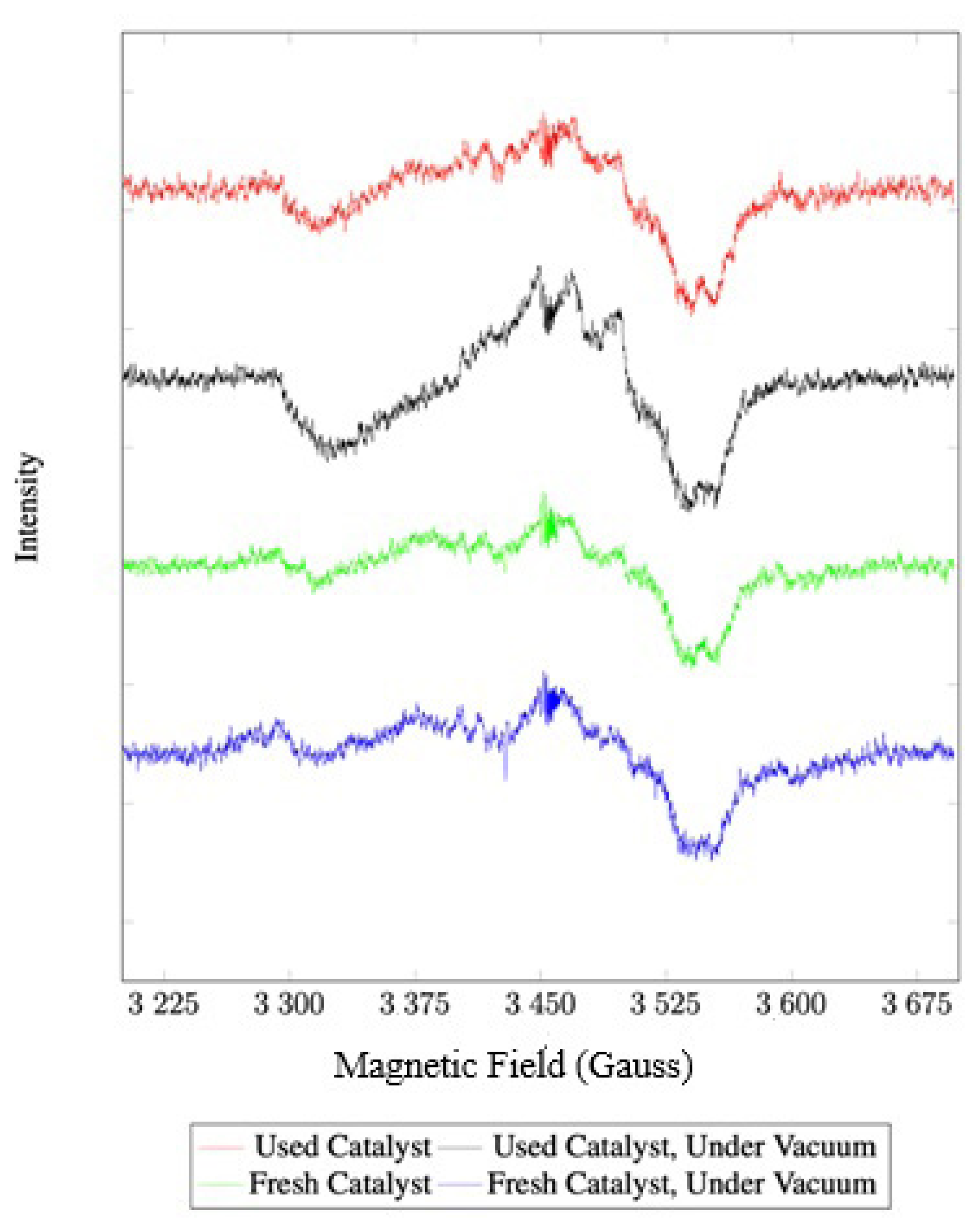

Supplement: Figure S3 — ESR spectra obtained from fresh and used catalyst. Measurements under vacuum are also shown. [file turkjchem-47-5-1285s3.tif]

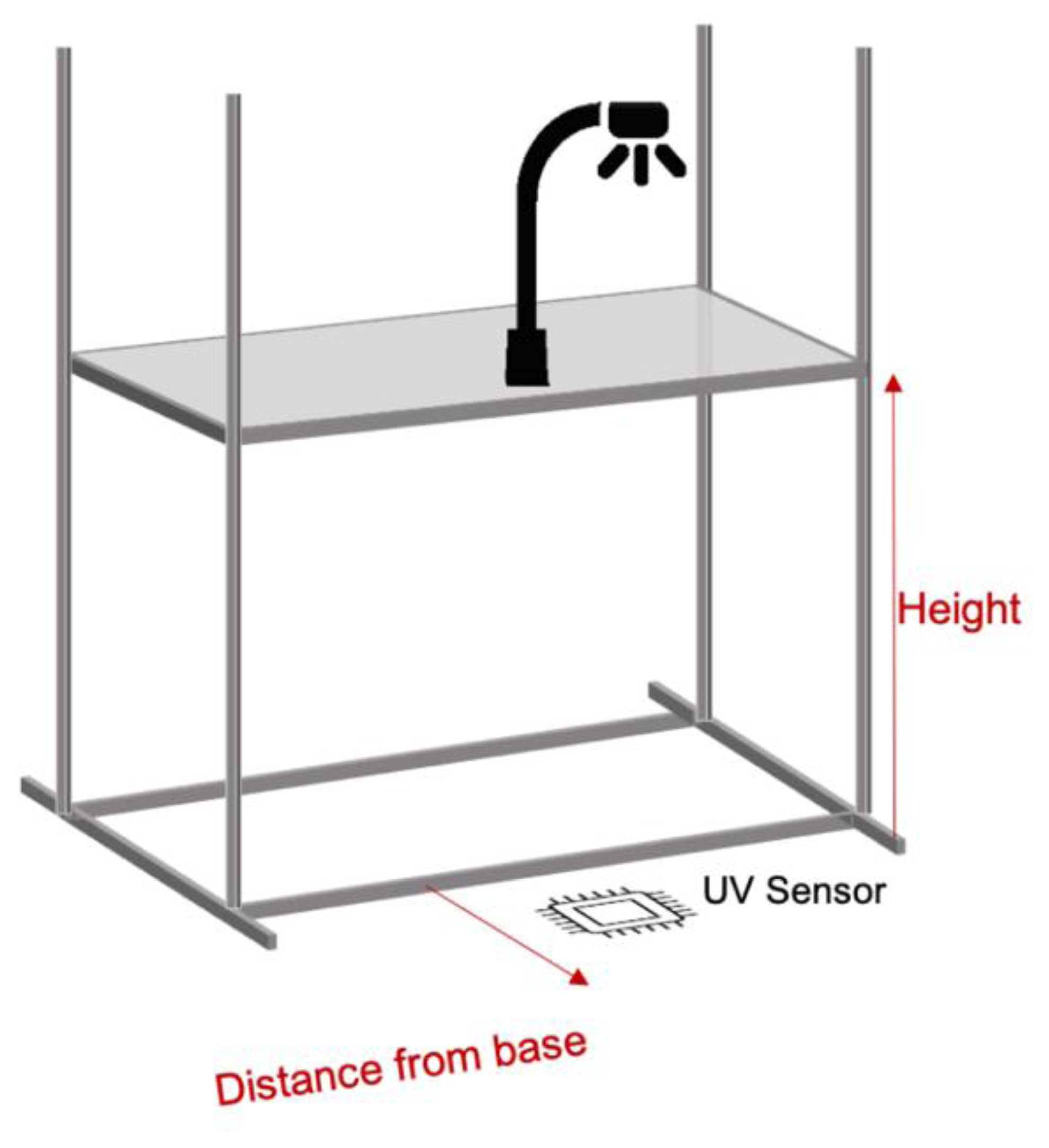

Supplement: Figure S4 — UV light intensity measurement set-up schematic [4]. [file turkjchem-47-5-1285s4.tif]

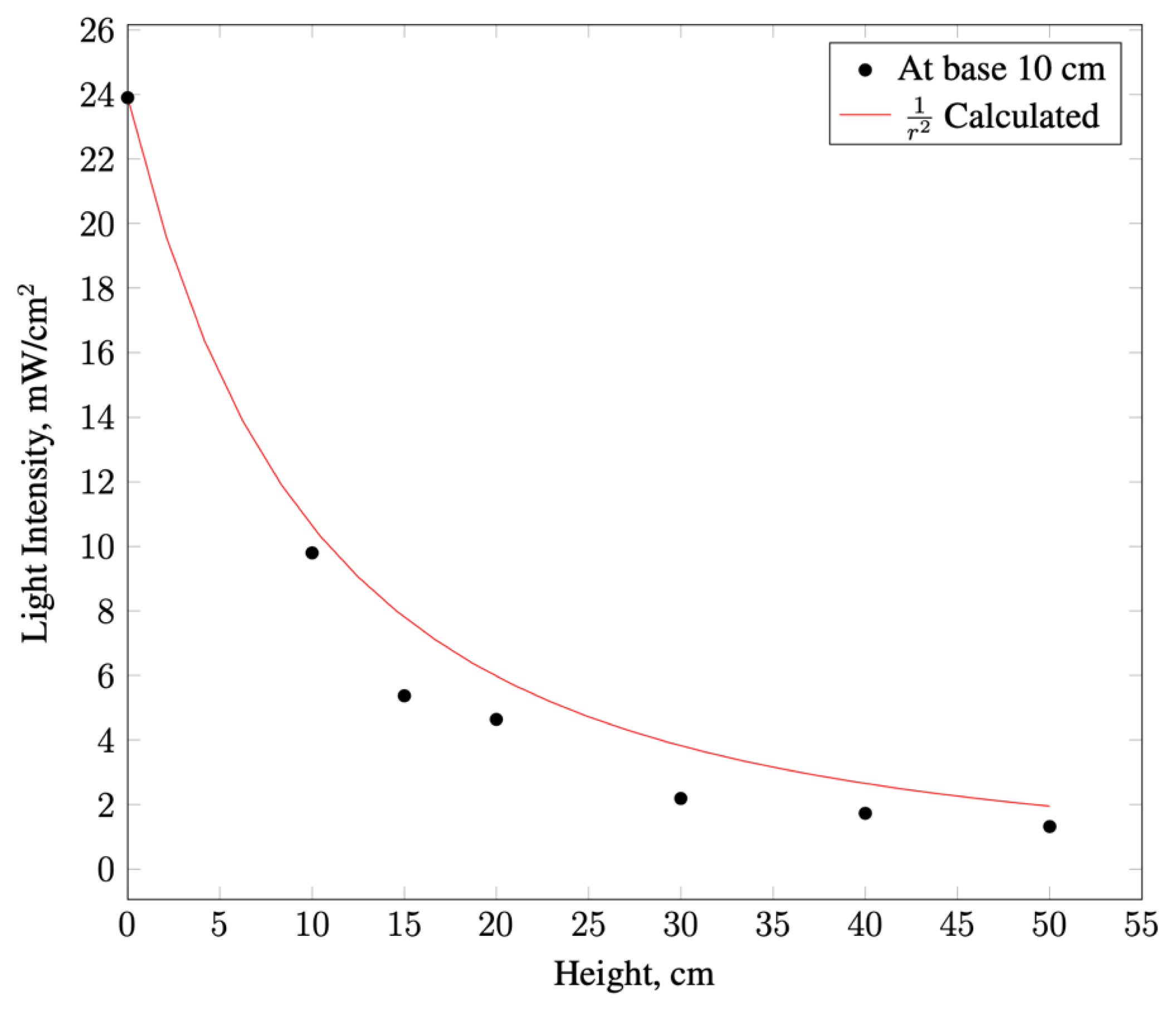

Supplement: Figure S5 — UV light intensity at base distance of 10 cm [4]. [file turkjchem-47-5-1285s5.tif]

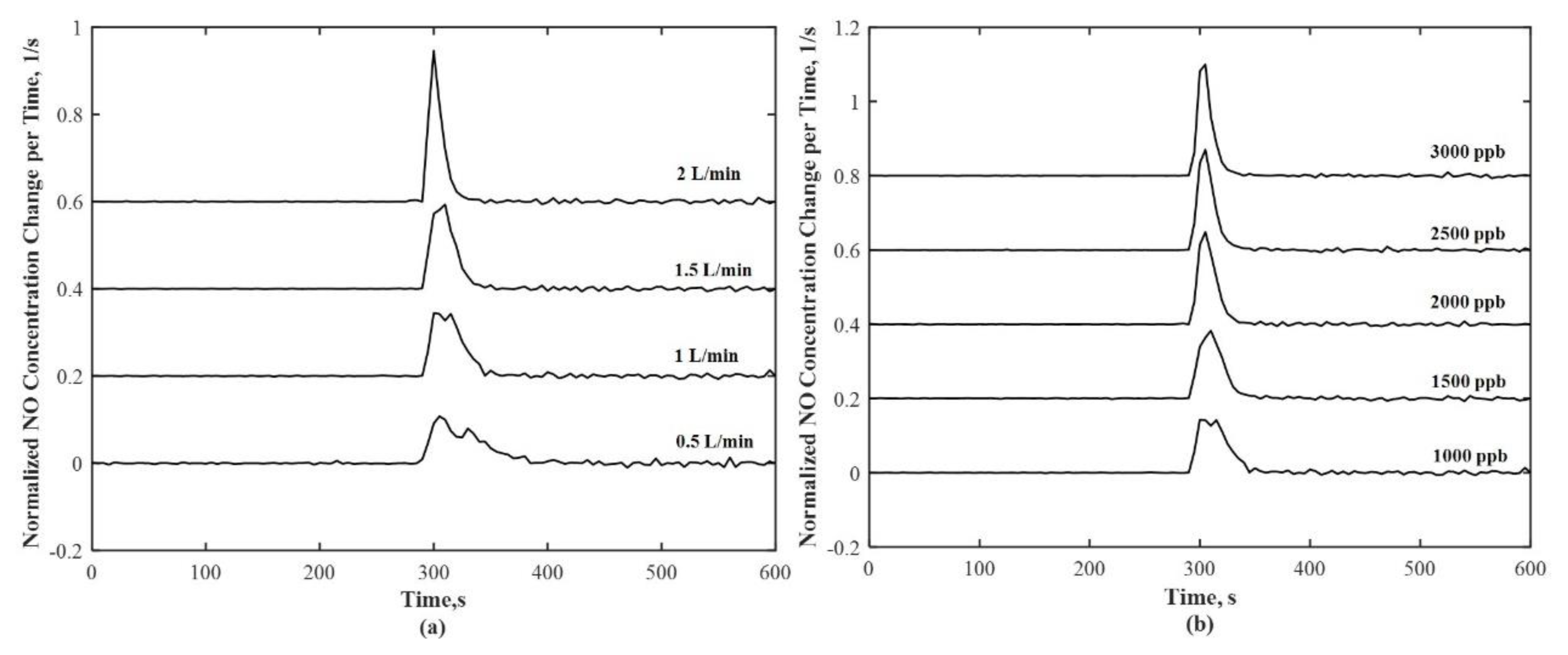

Supplement: Figure S6 — RTD analysis for various (a) tracer concentrations and (b) flow rates. [file turkjchem-47-5-1285s6.tif]

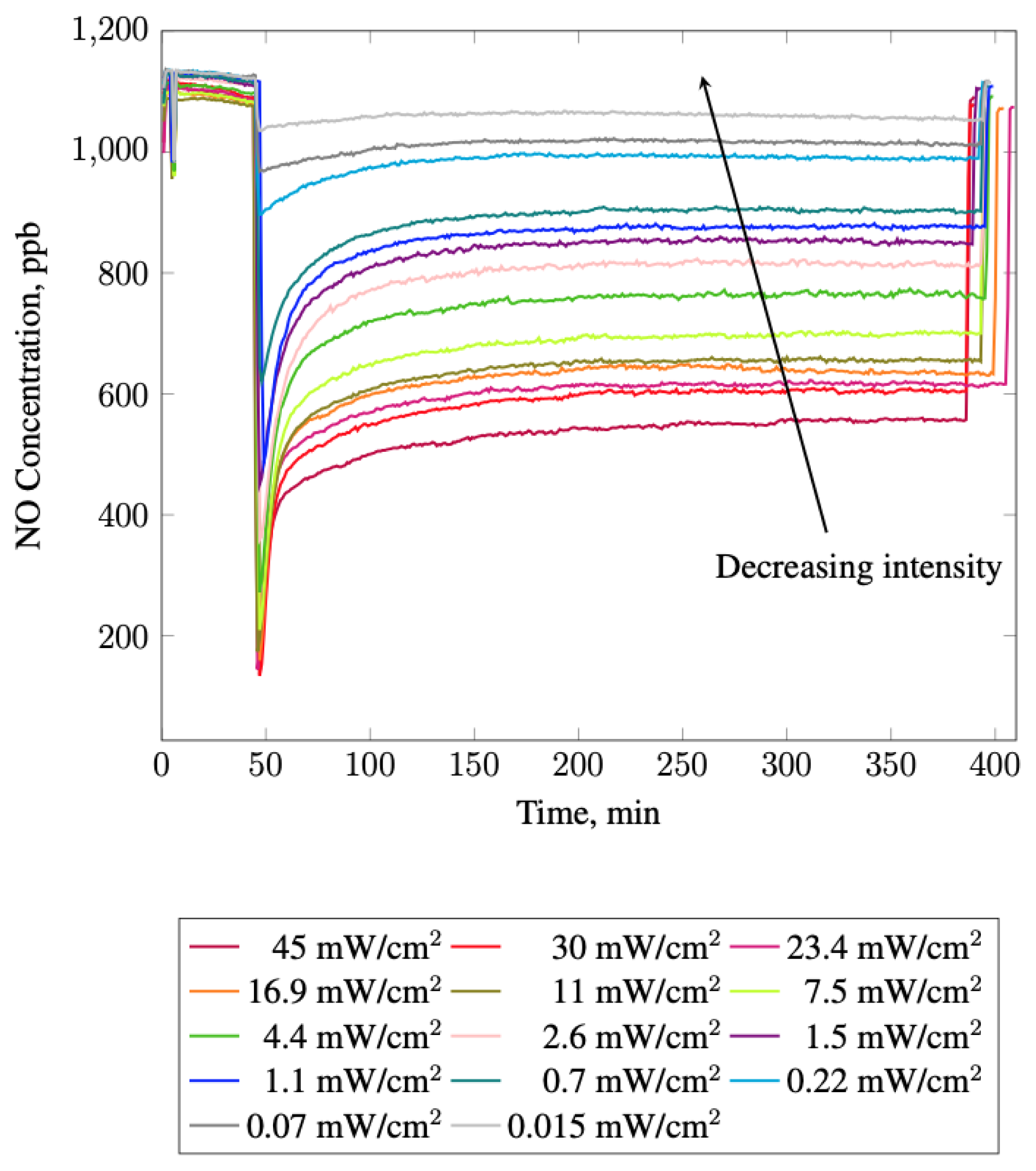

Supplement: Figure S7 — Effect of UV light intensity on NO oxidation raw data for the correlation shown in Figure 7. [file turkjchem-47-5-1285s7.tif]

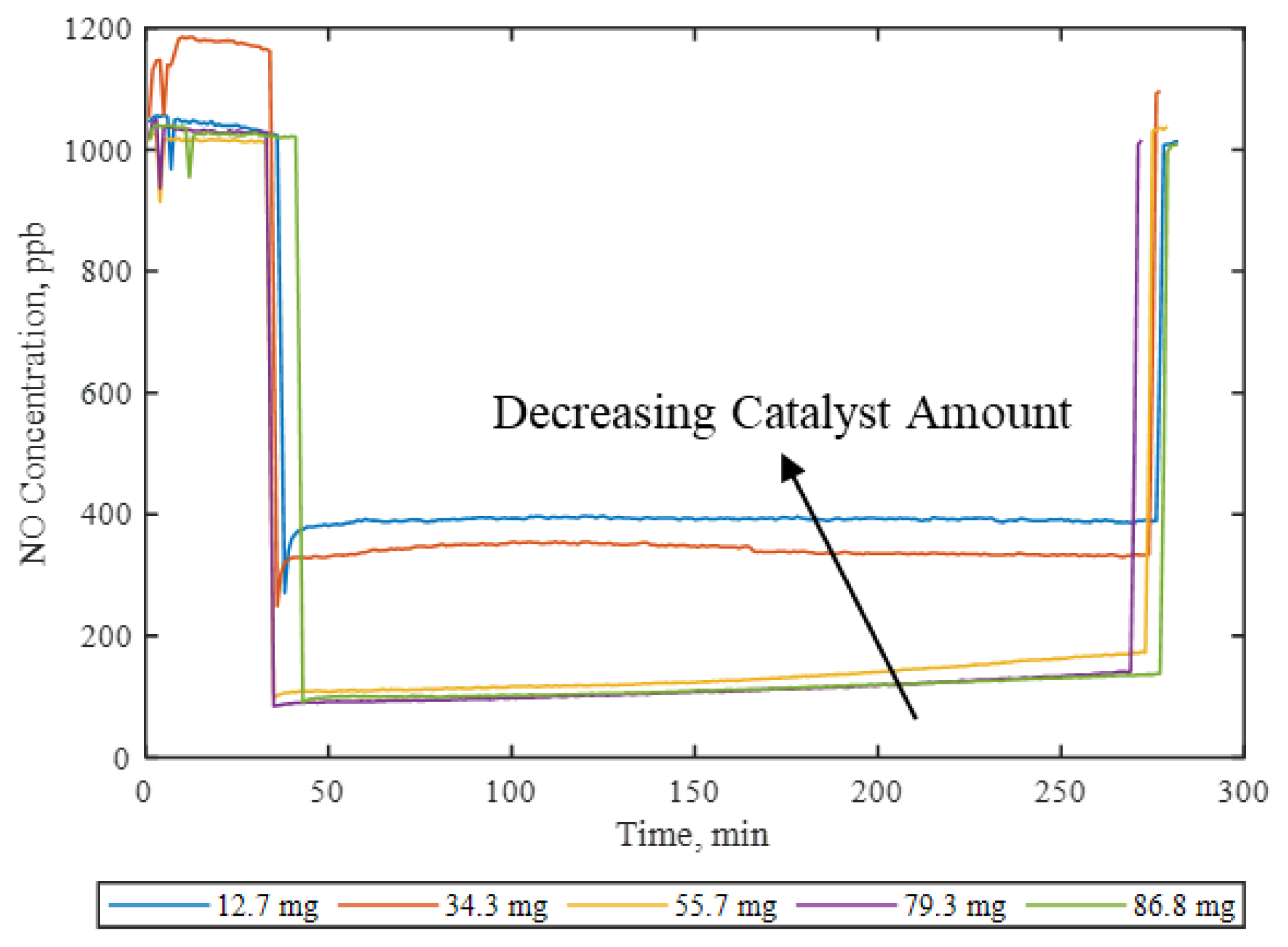

Supplement: Figure S8 — Effect of catalyst coating on NO oxidation at 45 mW/cm2. Raw data for the correlation shown in Figure 8. [file turkjchem-47-5-1285s8.tif]

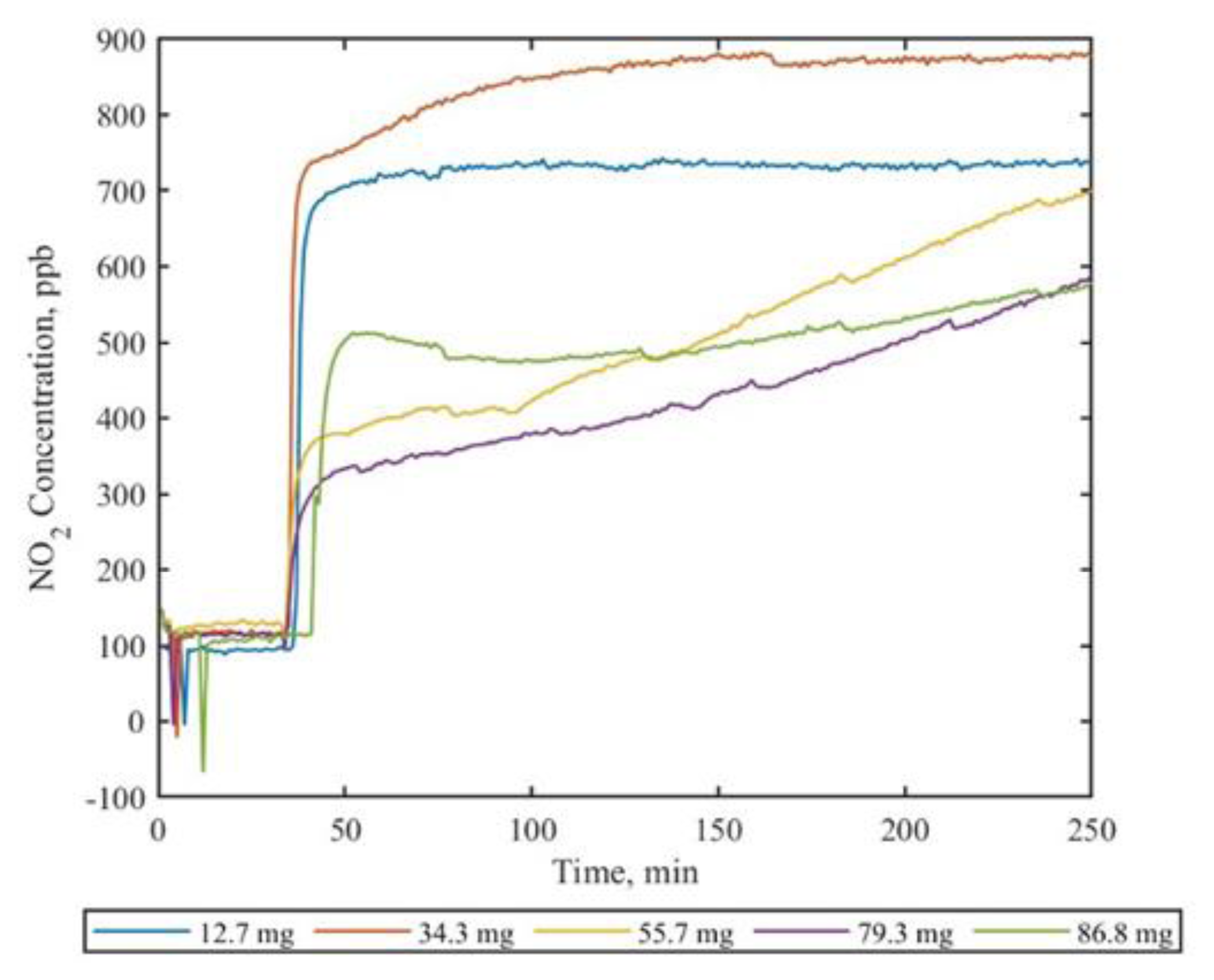

Supplement: Figure S9 — Effect of catalyst coating on NO2 formation at 45 mW/cm2. [file turkjchem-47-5-1285s9.tif]

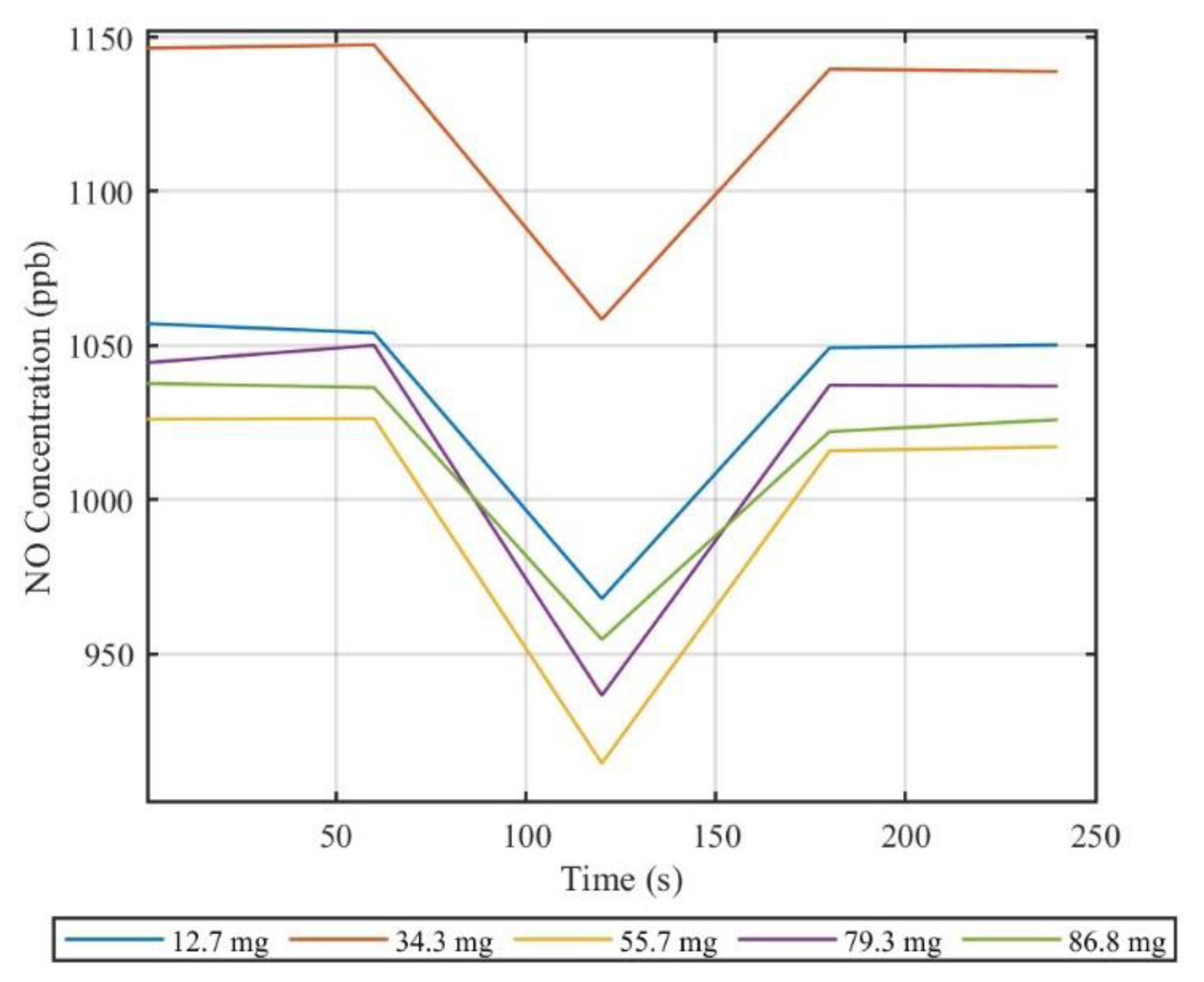

Supplement: Figure S10 — Effect of catalyst coating on first transient at 45 mW/cm2. Data were averaged every minute. It can be seen that peak intensities do not vary with catalyst amount. [file turkjchem-47-5-1285s10.tif]
